# HMGB1-Induced Cross Talk between PTEN and miRs 221/222 in Thyroid Cancer

**DOI:** 10.1155/2015/512027

**Published:** 2015-05-27

**Authors:** S. Mardente, E. Mari, I. Massimi, F. Fico, A. Faggioni, F. Pulcinelli, A. Antonaci, A. Zicari

**Affiliations:** ^1^Department of Experimental Medicine, Sapienza University of Rome, Viale Regina Elena 324, 00161 Rome, Italy; ^2^Department of Surgery, Sapienza University of Rome, Viale Regina Elena 324, 00161 Rome, Italy

## Abstract

High mobility group box 1 (HMGB1) is an ubiquitous protein that plays different roles in the nucleus, cytoplasm, and extracellular space. It is an important DAMP molecule that allows communication between damaged or tumor cells and the immune system. Tumor cells exploit HMGB1's ability to activate intracellular pathways that lead to cell growth and migration. Papillary thyroid cancer is a well-differentiated tumor and is often used to study relationships between cells and the inflammatory microenvironment as the latter is characterized by high levels of inflammatory cells and cytokines. Anaplastic thyroid cancer is one of the most lethal human cancers in which many microRNAs and tumor suppressor genes are deregulated. Upregulation of microRNAs 221 and 222 has been shown to induce the malignant phenotype in many human cancers via inhibition of PTEN expression. In this study we suggest that extracellular HMGB1 interaction with RAGE enhances expression of oncogenic cluster miR221/222 that in turn inhibits tumor suppressor gene PTEN in two cell lines derived from human thyroid anaplastic and papillary cancers. The newly identified pathway HMGB1/RAGE/miR221/222 may represent an effective way of tumor escape from immune surveillance that could be used to develop new therapeutic strategies against anaplastic tumors.

## 1. Introduction

MicroRNAs (miRNAs) can function as either oncogenes or tumor suppressor genes via regulation of cell proliferation or cell death. miRNAs 221 and 222 are two highly homologous microRNAs that have common targets [1, 2]. Among others, miR221 and miR222 are overexpressed in thyroid papillary cancer and in long term cell lines deriving from human papillary cancer [3, 4]. However, the cellular signaling and the way miRNAs 221 and 222 enhance growth have not been completely explained in thyroid tumorigenesis. We have recently demonstrated that inflammatory infiltrates present in papillary cancer are high in HMGB1 that may contribute to tumor transformation and escape immune surveillance [5–7].

HMGB1 is a DAMP molecule passively released by damaged cells and actively expressed by inflammatory cells [8, 9]. Within the different cellular compartments HMGB1 exerts different functions. Inside the nucleus it participates in DNA transcription, recombination, and repair directly or by increasing the p53, p57, and Rb affinity binding sites. In the cytoplasm it regulates autophagy and apoptosis by interacting with mitochondria and with heat shock proteins (HSPB1 and HSPB7). One of the roles of cytoplasmic HMGB1 is the detection of nucleic acids and enhancement of nucleic acid binding TLRs activation. Some TLRs such as TLR 9, 3, and 7 recognize various structures of RNAs and hypomethylated DNA [10].

Extracellular HMGB1 triggers inflammation by binding the multiligand receptor RAGE (the receptor for advanced glycation end products) and Toll-like receptors (TLR 2, TLR 4, and TLR 9) with signalling converge on the AKT activating pathways [11]. miRNAs 221 and 222 have been found to interact with the AKT pathway by inhibiting translation of the oncosuppressor PTEN [12].

The PTEN gene dephosphorylates phosphatidylinositol 3,4,5-trisphosphate (PIP3) leading to negative regulation of the protein kinase Akt. As well as nonprotein substrates (PIP3) PTEN might be able to act on protein substrates like focal adhesion kinases (FAK) and Shc and to control them. PTEN plays an important role not only in inducing apoptosis and cell cycle arrest in G1 but also in the regulation of cell adhesion and migration. Somatic deletions or mutations of this gene have been identified in many human cancers including glioblastomas and prostate and thyroid cancer [13, 14]. miRNA221 has been found in serum of thyroid cancer patients and this correlates with a negative prognosis [15–17]. miRNAs 221 and 222 have been found to regulate gastric carcinoma cell growth and radioresistance by suppressing the action of PTEN [18]. It has been lately demonstrated that circulating miRNAs signal through the Toll-like receptors TLR 7 and TLR 8 promoting inflammation and intercellular communication in the tumor microenvironment [19]. Anaplastic thyroid cancers are aggressive tumors [20] that fail to respond to radiation therapy. Besides hyperexpressing a set of miRNAs they frequently show mutations in PI3K kinase that phosphorylates AKT that in turn phosphorylates substrates involved in cell growth and survival. PTEN that negatively regulates the PI3K is also mutated in many cases of anaplastic and papillary cancer [21, 22].

In this study we analyzed the interactions between extracellular HMGB1 and the repressing action of the miRNAs 221/222 cluster on PTEN oncosuppressor mRNA that may lead to tumour progression.

## 2. Materials and Methods

### 2.1. Reagents

Anti-human PTEN monoclonal antibody was from DAKO (CA, USA). Anti-RAGE monoclonal antibody was from Millipore (CA).

Monoclonal anti-mouse IgG horseradish peroxidase conjugate was from GE Healthcare (UK). Human, recombinant HMGB-1 expressed in* E. coli* was from Sigma (Missouri, USA). miRIDIAN hairpin inhibitor, human hsa-miRNA221, hsa-miRNA222, and miRNA hairpin inhibitor control with Dy 547 and Dharmafect 1 transfection reagent were purchased from Thermo Fisher Scientific (Lafayette, CO, USA). Mature miRNA221 sequence was AGCUACAUUGUCUGCUGGGUUUC; mature miRNA222 sequence was CUCAGUAGCCAGUGUAGAUCCU.

TaqMan miRNA assays (Applied Biosystems, Foster City, CA, USA).

### 2.2. Cell Lines

The long term thyroid carcinoma cell lines, BCPAP and CAL62, obtained by DSMZ (BCPAP n. ACC 273, CAL62 n ACC 448) in October 2010 [23] were maintained in RPMI 1640 (GIBCO), supplemented with heat inactivated 10% FCS containing 2 mM L-glutamine, where required 10 nM HMGB1 was added to the cultures, at different times. Cell viability was determined by trypan blue exclusion test, where required (1 *μ*g/mL) anti-RAGE antibodies were added to the cultures for 2 h. Antibodies were removed by washings before the experiments. Cells were kept frozen in nitrogen liquid tanks (2 × 10^6^/1 mL FCS, 10% DMSO) and used between the 4th and 8th passage after revival.

### 2.3. Knockdown of miRNAs

AntagomiRs against miRNAs 221 and 222, and control oligonucleotide with DY 547, were obtained from Thermo Fisher Scientific (Lafayette, CO, USA). AntagomiRs were transiently transfected by using Dharmafect 1 transfection reagent (Thermo Fisher Scientific), into BCPAP and CAL62 cells, before treatment with HMGB1. Cells were seeded in 12-well plate at a density of 2 × 10^5^/well followed by an 18-hour incubation at 37°C before transfection. Then culture medium was replaced with 1 mL of antibiotic-free medium containing Dharmafect 1-reagent (2 *μ*L/mL) and the antagomiRs at 25 nM final concentration according to manufacturer instructions for 24 h. Controls with DY 547 and with Dharmafect reagent were included in the experiments as indicators for transfection efficiencies and negative controls, respectively. Transfection efficiency was determined by cytofluorimetric readings in the red light spectrum. In our experiments, transfection efficiency (DY 547 positive cells) reached 76%.

### 2.4. miRNA Relative Quantification by Real Time RT-PCR

Total RNA was extracted from BCPAP and CAL62 cells with Trizol reagent (Life technologies), according to the manufacturer's instructions. For the detection of mature miRNA221 and miRNA222, 50 ng of total RNA was reversely transcribed using High Capacity cDNA Archive Kit (Applied Biosystems, Foster City, CA, USA). Real time PCR was performed using a miRNA-specific TaqMan assay (Applied Biosystems) in Applied Biosystems 7900HT Sequence Detection System; amplifications were performed in triplicate and repeated twice. The ubiquitously expressed U6b small nuclear RNA (snRNA) was used for normalization. miRNAs amounts were quantified by using the ΔΔCt method for relative quantification of miRNAs expression.

### 2.5. Western Blot Analysis

Whole cell lysates were separated as previously described [6] on 12.5% SDS-polyacrylamide electrophoresis gel for PTEN. Samples were heat denatured for 5 min, loaded on standard Tris-HCl polyacrylamide gel, and run on ice at 40 V for the stacking gel and 80 V for the running gel. Proteins were transferred onto a previously activated PVDF membrane (Bio-Rad, Hercules). Membranes were then placed in TBS-T and 5% albumin for 1 h and probed overnight with the specific antibody at 4°C. At the end of incubation time, membranes were washed and incubated with anti-mouse IgG peroxidase conjugated secondary antibody (1 : 10000) for 1 h at room temperature. Membranes were stripped and incubated with anti-actin monoclonal antibody as a loading control. Signal was detected by autoradiography (Kodak Biomax) using the chemiluminescent peroxidase substrate kit (Sigma) and then quantified by densitometric analysis using quantity-one software (Bio-Rad).

### 2.6. Statistical Analysis

All determinations were carried out three times. Data are expressed as means ± SD. Data were analyzed by Student *t*-test. *p* < 0.05 was considered statistically significant.

## 3. Results

### 3.1. HMGB1 Increases Expression of miRNA221 and miRNA222 and Decreases Expression of PTEN in CAL62 and BCPAP Cells

HMGB1 is a DNA-binding nuclear protein that is released passively during cell death or actively following cytokine stimulation. It is abundantly found in the papillary tumor microenvironment where it has been shown to stimulate growth and chemotaxis of both immune and cancer cells. In order to investigate a possible mode of thyroid cancer cell proliferation induced by extracellular HMGB1, expression of miRNA221 and miRNA222 in CAL62 and BCPAP cells was assessed by PCR after addition of HMGB1 for 24, 48, and 72 h. The concentration of 10 nM HMGB1 was chosen according to our previous studies [3] and to internal dose-response controls performed on different cell lines and in primary cultures obtained from papillary cancer and nonneoplastic lesions (6).

**Figure 1:**
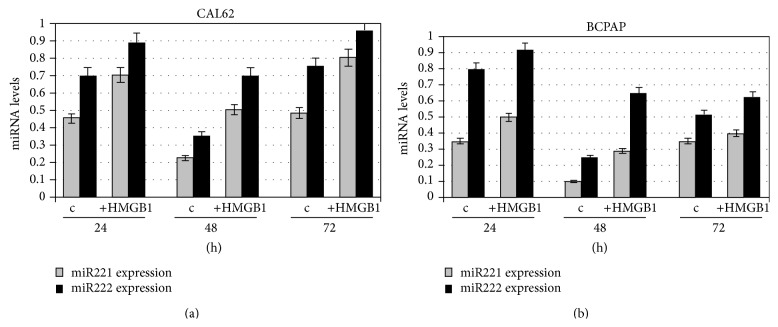
Expression of miR221 and miR222 in CAL62 and BCPAP cells before and after treatment with HMGB1. CAL62 cells (a) and BCPAP cells (b) were cultured for 24, 48, and 72 h, with or without 10 nM HMGB1 which was added to the culture medium. Expression levels of miRs were detected by real time RT-PCR. miRNAs amounts were quantified by using the ΔΔCt method and normalized with U6b small nuclear RNA (snRNA) expression. Histograms represent the mean values of 3 independent experiments (±SD).

Figure 1 shows that treatment with HMGB1 increases expression of miRNAs 221 and 222 significantly in both cell lines (*p* < 0.01).

**Figure 2:**
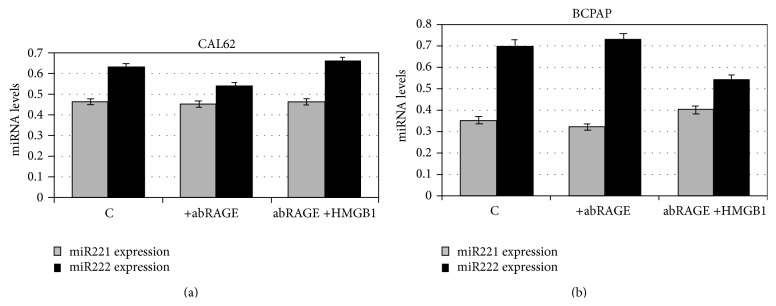
Expression of miR221 and miR222 in anti-RAGE pretreated CAL62 and BCPAP cells with or without HMGB1 for 24 h. CAL62 (a) and BCPAP cells (b) were treated with anti-RAGE antibody and then cultured with 10 nM HMGB1 for 24 h. Expression levels of mirR 221 and 222 were detected by RT-PCR and quantified with the ΔΔCt method. Histograms represent the mean values of 3 independent experiments (±SD).

In order to show if the above effect was mediated by HMGB1-RAGE binding, cells were treated with anti-RAGE antibody and then stimulated with HMGB1 for 24 h.  Figure 2 shows that when RAGE is blocked by anti-RAGE, the presence of HMGB1 does not significantly increase miR221 and miR222 expression in both CAL62 cells and BCPAP. Overall the results indicate that induction of miR221 and miR222, the latter at a minor extent, is RAGE dependent.

At the light of HMGB1 effect played on miRNAs 221 and 222 expression and considering that they have been shown to inhibit PTEN mRNA in many human cancers, we studied the expression of PTEN after treatment of cells with HMGB1 for up to 72 h.

**Figure 3:**
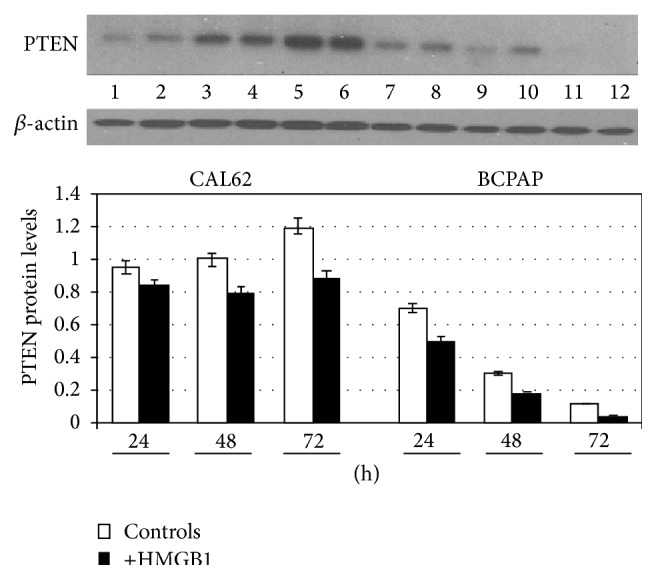
Effect of HMGB1 on PTEN expression in CAL62 and BCPAP cells. CAL62 and BCPAP cells were cultured for 24, 48, and 72 h, with or without 10 nM HMGB1 which was added to the culture medium. Expression levels of protein PTEN 55 kDa was detected by western blot in whole cell lysates (*p* < 0.05). Representative experiment of 3 different western blots.

Interestingly PTEN protein levels shown in Figure 3 constantly decrease after addition of HMGB1 in both CAL62 and BCPAP cells compared with the controls (*p* < 0.05).

When RAGE receptors are blocked by anti-RAGE antibodies, HMGB1 fails to induce PTEN decrease as demonstrated by cytofluorimetric analysis shown in Supplementary Figure  1 (see Supplementary Material available online at http://dx.doi.org/10.1155/2015/512027).

### 3.2. AntagomiRs 221 and 222 Increase Expression of PTEN in CAL62 and BCPAP Cells

In order to demonstrate that miRNA221 and miRNA222 were linked with PTEN function in CAL62 and BCPAP cells we delivered specific antagomiRs and determined miRs expression.

**Figure 4:**
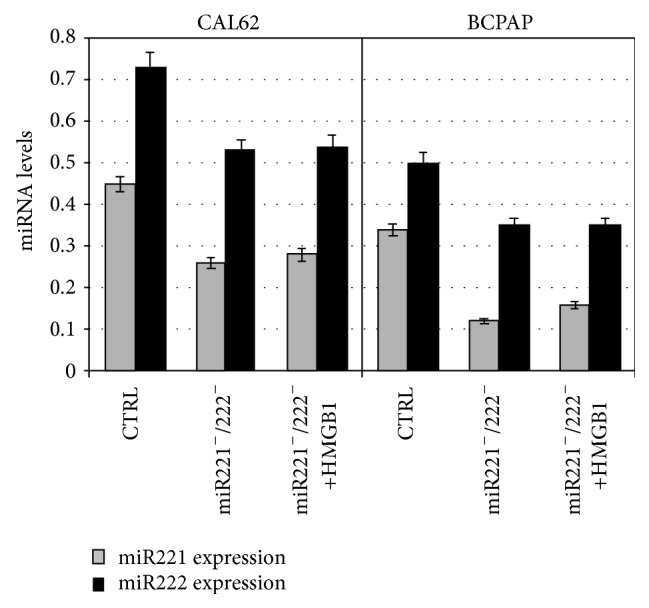
Expression of miR221 and miR222 after transfection with antagomiRs 221/222 and in transfectants after treatment with HMGB1. Q-RT-PCR analysis of miR221 and miR222 level expression in CAL62 and BCPAP cells transfected with miR221 and miR222 antagonists (transfectants: miR221^−^/miR222^−^) or negative control (CTR), at 25 nM final concentration for 24 h, where indicated transfectants were treated with 10 nM HMGB1 for 24 h. Data were normalized with U6b small nuclear RNA (snRNA) expression and reported as mean ± SD of 3 experiments (*p* < 0.01).

Figure 4 shows that cotransfection of antagomiRs 221 and 222 in CAL62 was able to reduce expression of miRNA221 by 44.5% and miRNA222 by 25%. Reduction of miR221 and miR222 expression in BCPAP cells was, respectively, of 66.6% and 30%. In order to show that HMGB1 was not able to increase miRs expression when miRs were blocked by antagomiRs we treated transfectant cells with HMGB1 for 24 h and obtained a similar reduction to that obtained in transfectant cells without HMGB1 treatment (Figure 4). This result shows that HMGB1 is responsible for miR221/222 overexpression.

**Figure 5:**
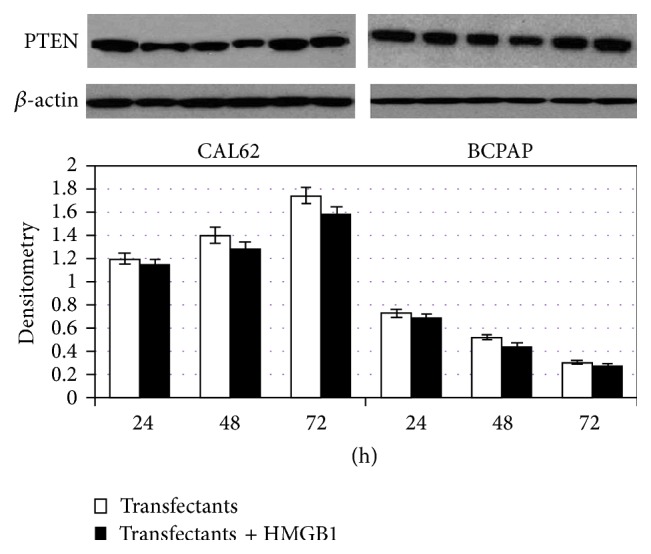
PTEN expression in CAL62 and BCPAP cells transfected with antagomiRs 221/222 and then treated or not with HMGB1. Effect of antagomiRs 221 and 222 on PTEN expression. CAL62 and BCPAP cells were transfected with antagomiRs 221 and 222 (transfectants). 24 h after transfection, cells were cultured for 24, 48, and 72 h with or without the addition of 10 nM HMGB1. At the end of incubation times, the cell lysates were harvested and subjected to western blot analysis for PTEN protein expression. *p* < 0.01 versus parental cells and transfectants; *p* < 0.01 versus parental cells and transfectants with HMGB1; transfectants versus transfectants with HMGB1 were not statistically significative. Representative experiment of 3 different western blots.

The next set of experiments was designed to show if the action of HMGB1 on miR221/222 cluster was functional on PTEN, which is a known target of miR221 and miR222. For this reason, transfectant cells were grown for up to 72 h in presence of 10 nM HMGB1 and PTEN expression was determined by western blot. As shown in Figure 5, HMGB1 did not significantly reduce PTEN expression in transfectants indicating that it interacts with PTEN through the miRNAs 221/222 cluster.

### 3.3. HMGB1 Increases Growth of CAL62 and BCPAP Cells While AntagomiRs 221 and 222 Inhibit It

**Figure 6:**
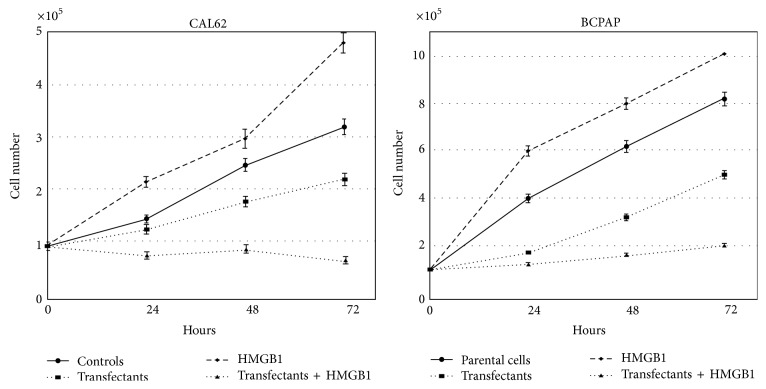
Effect of HMGB1 on the proliferation rate of parental and transfected CAL62 and BCPAP cells. 10^5^ parental cells and transfectants were seeded into a 6-well plate at time 0 with or without 10 nM HMGB1. At the indicated times cell number and cell viability of each well were counted with a haemocytometer. Data represent the mean values of 3 independent experiments (±SD). *p* < 0.01 versus transfectants and parental cells and versus transfectants and transfectants treated with HMGB1.

Figure 6 shows growth curves of both parental and transfectant CAL62 and BCPAP cells. Addition of 10 nM HMGB1 to CAL62 cells for 24, 48, and 72 h increases growth in a significative way. A similar trend but a much higher increase was obtained in BCPAP cells cultured in presence of HMGB1.

CAL62 cells transfected with antagomiRs 221 and 222 show a time dependent decrease of growth (from 13.4% at 24 h to 31.2% at 72 h) compared with controls. HMGB1 added to the CAL62 and BCPAP transfectants reduces growth, respectively, of 38.5% and 42% at 24 h, 50% and 48.6% at 48 h, and 68% and 60% at 72 h. The decrease of growth in transfected cells is due to the high rate of cell death induced by the blockage of miR221/222. This has been reported in many cell lines [18]. Interestingly HMGB1 induces a higher rate of death into transfected cells compared with parental cells (shown in Supplementary Figure  2).

## 4. Discussion

Following our previous study demonstrating that HMGB1 increases miR221 and miR222 expression in short term primary cultures of papillary cancer cells compared with normal cells obtained from contralateral thyroid lobe and with cells from patients with nonneoplastic lesions, we report here that HMGB1 induces miRNAs 221 and 222 hyperexpression in both differentiated (BCPAP) and anaplastic (CAL62) thyroid cancer cell lines. In turn miR221/222 cluster inhibits the tumor suppressive role of PTEN. We are reporting here for the first time that exogenous HMGB1 decreases PTEN expression in thyroid cell lines.

It is known that HMGB1 proteins are involved in transcription regulation through many different epigenetic processes [24]. In a variety of human tumors elevated levels of HMGs are associated with a poor prognosis and with tumor invasion and metastasis [25]. When HMGB1 proteins are released in the tumor microenvironment by immune cells, they act as both autocrine and paracrine messengers. They bind TLR 2, TLR 4, and RAGEs present on immune cells and on tumor cells [26, 27] and activate NF*κ*B, PI3K/Akt, and other activating pathways. It is known that miRNAs 221 and 222 enhance Akt phosphorylation through downregulation of PTEN in many human cancers [28].

HMGB1 is required for the immune response to nucleic acids by activating intracytoplasmic TLR 3, 7, and 9. An interesting colocalization of HMGB1 and TLR 9 with an endosomal marker EEA1 has been shown in quiescent cells upon stimulation with oligodeoxynucleotides (CpG-ODN) [29] meaning that cytoplasmic HMGB1 is able to interact with nucleotides. HMGB1 has been shown to promote proliferation and migration of different tumor cells via its known receptor TLR 4 [30]. We are reporting here that HMGB1 action on both BCPAP and CAL62 cells is specific and is due to RAGEs intracellular pathways. The blocking action of anti-RAGE antibody prevents both PTEN reduction and increase of cellular proliferation (not shown). The data we obtained with cells transfected with antagomiRs 221 and 222 and treated with HMGB1 suggest that stimulation of RAGEs by HMGB1 leads to inactivation of oncosuppressor PTEN through an interaction with miRNAs 221/222. The inactivity of miRNAs was able to block HMGB1 related growth increase, meaning that blocking miRNAs 221 and 222 overrides HMGB1 effects on PTEN expression and cell growth. To our knowledge this is the first time it has been shown that the newly identified pathway HMGB1/RAGE/miR221/miR222 may connect immune system with cancer. Although we have been able to establish that RAGE signaling can regulate the expression of miR221/222, interactions between intracellular RAGE signaling and miR221/222 require further investigation.

## 5. Conclusions

Our results may help the finding of new therapeutic strategies for the treatment of anaplastic thyroid cancer and they may provide new prognostic factors that could address the right therapeutic strategy in papillary cancer.

## Supplementary Material

Figure 1 supplementary: Effect of RAGE blockage on PTEN expression in CAL 62 and BC PAP cells
Cytofluorimetric analysis of CAL 62 and BC PAP cells cultured for 24 h with or without HMGB1. Where indicated cells were pre-treated with anti RAGE antibody. Histograms represent log fluorescence versus cell number, gated on cell population of a side scatter/forward scatter histogram (SS/FS). Cell number is indicated on the y axis and fluorescence intensity is represented on the x axis. Cursor B indicates cell specific fluorescence; percentages of positive cells are reported in each histogram. Aspecific fluorescence was gated with samples labelled with secondary antibodies (monoclonal anti-mouse IgG) (not shown). Representative experiment.
Figure 2 Supplementary: Cell viability in CAL 62 and BC PAP cells treated or not with HMGB1
%% of dead cells in parental CAL 62 and BC PAP cells. 10 5 parental and transfected cells were seeded into a 6 well plate. Where indicated 10 nM HMGB1 was added to the cultures. Dead cells were determined with the trypan bleu exclusion test. Data represent the mean values of 3 independent experiments (+ SD). p<0.01 versus transfectants and parental cells and <0.05 versus transfectants and transfectants treated with HMGB1.

## References

[1] Garofalo M., Quintavalle C., Romano G., Croce C. M., Condorelli G. (2012). MiR221/222 in cancer: their role in tumor progression and response to therapy. *Current Molecular Medicine*.

[2] Bi C., Chng W. J. (2014). MicroRNA: important player in the pathobiology of multiple myeloma. *BioMed Research International*.

[3] Mardente S., Zicari A., Consorti F. (2010). Cross-talk between NO and HMGB1 in lymphocytic thyroiditis and papillary thyroid cancer. *Oncology Reports*.

[4] Hui A., How C., Ito E., Liu F.-F. (2011). Micro-RNAs as diagnostic or prognostic markers in human epithelial malignancies. *BMC Cancer*.

[5] Cunha L. L., Marcello M. A., Ward L. S. (2014). The role of the inflammatory microenvironment in thyroid carcinogenesis. *Endocrine-Related Cancer*.

[6] Mardente S., Mari E., Consorti F. (2012). HMGB1 induces the overexpression of miR-222 and miR-221 and increases growth and motility in papillary thyroid cancer cells. *Oncology Reports*.

[7] Korbelik M., Zhang W., Merchant S. (2011). Involvement of damage-associated molecular patterns in tumor response to photodynamic therapy: surface expression of calreticulin and high-mobility group box-1 release. *Cancer Immunology, Immunotherapy*.

[8] Tsung A., Tohme S., Billiar T. R. (2014). High-mobility group box-1 in sterile inflammation. *Journal of Internal Medicine*.

[9] Dong X. D., Ito N., Lotze M. T. (2007). High mobility group box I (HMGB1) release from tumor cells after treatment: implications for development of targeted chemoimmunotherapy. *Journal of Immunotherapy*.

[10] Drexler S. K., Foxwell B. M. (2010). The role of toll-like receptors in chronic inflammation. *The International Journal of Biochemistry & Cell Biology*.

[11] Yu M., Wang H., Ding A. (2006). HMGB1 signals through toll-like receptor (TLR) 4 and TLR2. *Shock*.

[12] Leslie N. R., Spinelli L., Tibarewal P. (2010). Indirect mechanisms of carcinogenesis via downregulation of PTEN function. *Advances in Enzyme Regulation*.

[13] Antico Arciuch V. G., Russo M. A., Dima M. (2011). Thyrocyte-specific inactivation of p53 and Pten results in anaplastic thyroid carcinomas faithfully recapitulating human tumors. *Oncotarget*.

[14] Guigon C. J., Zhao L., Willingham M. C., Cheng S.-Y. (2009). PTEN deficiency accelerates tumour progression in a mouse model of thyroid cancer. *Oncogene*.

[15] Pallante P., Visone R., Croce C. M., Fusco A. (2010). Deregulation of microRNA expression in follicular cell-derived human thyroid carcinomas. *Endocrine-Related Cancer*.

[16] Yu S., Liu Y., Wang J. (2012). Circulating microRNA profiles as potential biomarkers for diagnosis of papillary thyroid carcinoma. *Journal of Clinical Endocrinology and Metabolism*.

[17] Lassalle S., Hofman V., Ilie M. (2011). Can the microRNA signature distinguish between thyroid tumors of uncertain malignant potential and other well-differentiated tumors of the thyroid gland?. *Endocrine-Related Cancer*.

[18] Chun-zhi Z., Lei H., An-ling Z. (2010). MicroRNA-221 and microRNA-222 regulate gastric carcinoma cell proliferation and radioresistance by targeting PTEN. *BMC Cancer*.

[19] He W. A., Calore F., Londhe P., Canella A., Guttridge D. C., Croce C. M. (2014). Microvesicles containing miRNAs promote muscle cell death in cancer cachexia via TLR7. *Proceedings of the National Academy of Sciences of the United States of America*.

[20] Smallridge R. C., Marlow L. A., Copland J. A. (2009). Anaplastic thyroid cancer: molecular pathogenesis and emerging therapies. *Endocrine-Related Cancer*.

[21] Liu Z., Hou P., Ji M. (2008). Highly prevalent genetic alterations in receptor tyrosine kinases and phosphatidylinositol 3-kinase/Akt and mitogen-activated protein kinase pathways in anaplastic and follicular thyroid cancers. *Journal of Clinical Endocrinology and Metabolism*.

[22] Arlot-Bonnemains Y., Baldini E., Martin B. (2008). Effects of the Aurora kinase inhibitor VX-680 on anaplastic thyroid cancer-derived cell lines. *Endocrine-Related Cancer*.

[23] Pilli T., Prasad K. V., Jayarama S., Pacini F., Prabhakar B. S. (2009). Potential utility and limitations of thyroid cancer cell lines as models for studying thyroid cancer. *Thyroid*.

[24] Yoshida M., Ueda T. (2010). HMGB proteins and transcriptional regulation. *Biochimica et Biophysica Acta—Gene Regulatory Mechanisms*.

[25] D’Angelo D., Mussnich P., Rosa R., Bianco R., Tortora G., Fusco A. (2014). High mobility group A1 protein expression reduces the sensitivity of colon and thyroid cancer cells to antineoplastic drugs. *BMC Cancer*.

[26] Fabbri M., Paone A., Calore F. (2012). MicroRNAs bind to Toll-like receptors to induce prometastatic inflammatory response. *Proceedings of the National Academy of Sciences of the United States of America*.

[27] Ma J., Liu J., Wang Z. (2014). NF-kappaB-dependent MicroRNA-425 upregulation promotes gastric cancer cell growth by targeting PTEN upon IL-1*β* induction. *Molecular Cancer*.

[28] Muniyan S., Ingersoll M. A., Batra S. K., Lin M. (2014). Cellular prostatic acid phosphatase, a PTEN-functional homologue in prostate epithelia, functions as a prostate-specific tumor suppressor. *Biochimica et Biophysica Acta*.

[29] Ivanov S., Dragoi A. M., Wang X. (2007). Anovel role for HMGB1 in TLR9-mediated inflammatory responses to CpG-DNA. *Blood*.

[30] Wang F.-P., Li L., Li J., Wang J.-Y., Wang L.-Y., Jiang W. (2013). High mobility group box-1 promotes the proliferation and migration of hepatic stellate cells via TLR4-dependent signal pathways of PI3K/Akt and JNK. *PLoS ONE*.

