# Supplementary material for: HMGB1-Induced Cross Talk between PTEN and miRs 221/222 in Thyroid Cancer
Source: Biomed Res Int. 2015 May 27;2015:512027. doi: 10.1155/2015/512027 (PMC4461734; doi:10.1155/2015/512027)
Supplement: Supplementary file 1 — Figure 1 supplementary: Effect of RAGE blockage on PTEN expression in CAL 62 and BC PAP cells Cytofluorimetric analysis of CAL 62 and BC PAP cells cultured for 24 h with or without HMGB1. Where indicated cells were pre-treated with anti RAGE antibody. Histograms represent log fluorescence versus cell number, gated on cell population of a side scatter/forward scatter histogram (SS/FS). Cell number is indicated on the y axis and fluorescence intensity is represented on the x axis. Cursor B indicates cell specific fluorescence; percentages of positive cells are reported in each histogram. Aspecific fluorescence was gated with samples labelled with secondary antibodies (monoclonal anti-mouse IgG) (not shown). Representative experiment. Figure 2 Supplementary: Cell viability in CAL 62 and BC PAP cells treated or not with HMGB1 %% of dead cells in parental CAL 62 and BC PAP cells. 10 5 parental and transfected cells were seeded into a 6 well plate. Where indicated 10 nM HMGB1 was added to the cultures. Dead cells were determined with the trypan bleu exclusion test. Data represent the mean values of 3 independent experiments (+ SD). p<0.01 versus transfectants and parental cells and <0.05 versus transfectants and transfectants treated with HMGB1. [file 512027.f1.pdf]

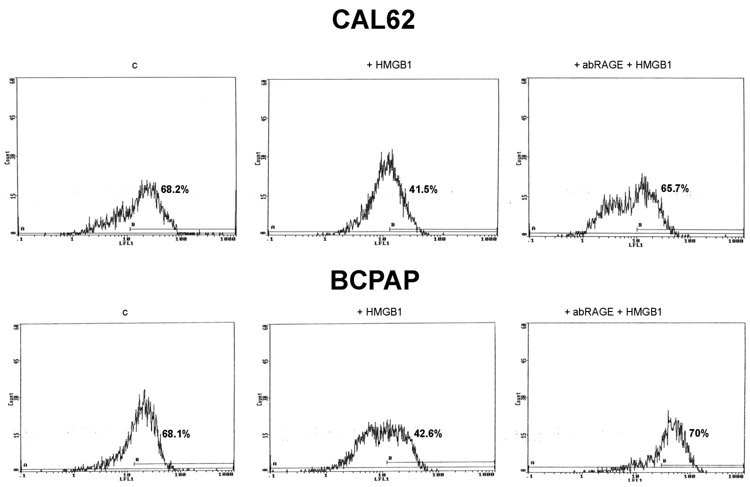

Fig.1 supplementary: Effect of RAGE blockage on PTEN expression in CAL62 and BCPAP cells

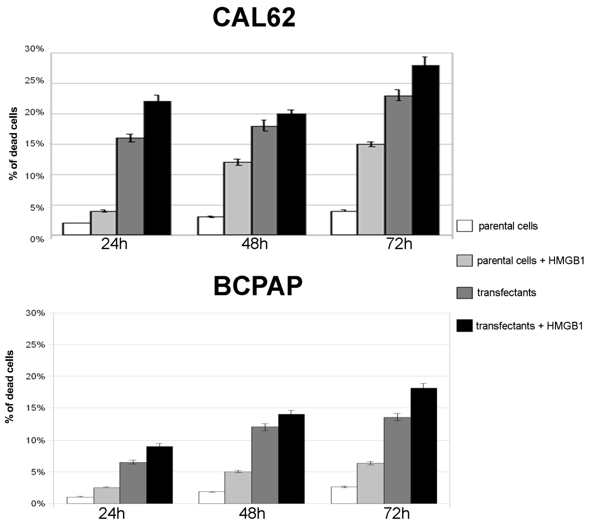

Fig 2 supplementary:Cell viability in CAL 62 and BCPAP cells treated or not with HMGB1
